# Glenohumeral Joint Kinematics following Clavicular Fracture and Repairs

**DOI:** 10.1371/journal.pone.0164549

**Published:** 2017-01-06

**Authors:** Claudio Rosso, Michael Nasr, Kempland C. Walley, Ethan R. Harlow, Babak Haghpanah, Ashkan Vaziri, Arun J. Ramappa, Ara Nazarian, Joseph P. DeAngelis

**Affiliations:** 1 Center for Advanced Orthopaedic Studies, Carl J. Shapiro Department of Orthopaedic Surgery, Beth Israel Deaconess Medical Center, Harvard Medical School, Boston, Massachusetts, United States of America; 2 Department of Mechanical and Industrial Engineering, Northeastern University, Boston, Massachusetts, United States of America; 3 Carl J. Shapiro Department of Orthopaedic Surgery, Beth Israel Deaconess Medical Center, Harvard Medical School, Boston, Massachusetts, United States of America; University of Zaragoza, SPAIN

## Abstract

**Background:**

The purpose of this biomechanical study was to determine the effect of shortened clavicle malunion on the center of rotation of the glenohumeral (GH) joint, and the capacity of repair to restore baseline kinematics.

**Methods:**

Six shoulders underwent automated abduction (ABD) and abbreviated throwing motion (ATM) using a 7-DoF automated upper extremity testing system in combination with an infrared motion capture system to measure the center of rotation of the GH joint. ATM was defined as pure lateral abduction and late cocking phase to the end of acceleration. Torsos with intact clavicle underwent testing to establish baseline kinematics. Then, the clavicles were subjected to midshaft fracture followed by kinematics testing. The fractured clavicles underwent repairs first by clavicle length restoration with plate fixation, and then by wiring of fragments with a 2-cm overlap to simulate shortened malunion. Kinematic testing was conducted after each repair technique. Center of rotation of the GH joint was plotted across all axes to outline 3D motion trajectory and area under the curve.

**Results:**

Throughout ABD, malunion resulted in increased posterior and superior translation compared to baseline. Plate fixation restored posterior and superior translations at lower abduction angles but resulted in excess anterior and inferior translation at overhead angles. Throughout ATM, all conditions were significantly anterior and superior to baseline. Translation with malunion was situated anterior to the fractured and ORIF conditions at lower angles of external rotation. Plate fixation did not restore baseline anteroposterior or superoinferior translation at any angle measured.

**Conclusions:**

This study illustrates the complex interplay of the clavicle and the GH joint. While abnormal clavicle alignment alters shoulder motion, restoration of clavicle length does not necessarily restore GH kinematics to baseline. Rehabilitation of the injured shoulder must address the osseous injury and the dynamic forces of the shoulder girdle.

## Introduction

Clavicle fractures account for approximately 2–10% of all fractures, with the majority occurring in men between the ages of 13 and 20 years [1,2]. Their bimodal distribution has a peak below the age of 25 and another among the elderly [3]. While the Allman classification has been used to describe clavicle fractures, studies have shown a predilection for fractures involving the middle third [1,4]. The sternoclavicular and acromioclavicular ligaments support the medial and lateral clavicle, respectively, leaving the thinner, less medullous middle third vulnerable to fracture [5].

Displacement is a common consequence of midshaft clavicle fractures, occurring in up to 73% of cases [1,6,7]. While risk of nonunion is relatively low after proper assessment of known risk factors (extensive displacement, comminution, shortening, etc.), malunions are common because gravity and muscle forces twist and shorten the healing clavicle into an aesthetic and functional anomaly [6]. Non-operative treatments forego the need for surgeries that carry the risk of intraoperative and postoperative complications; maintaining alignment and preventing malunion with closed reduction, however, is virtually impossible [1,6].

Previous studies have shown that shortening of more than 15–20 millimeters (mm) results in a symptomatic malunion and patient dissatisfaction [8–12]. Additionally, studies have revealed that patients with a foreshortened clavicle malunion have decreased shoulder function with loss of strength [13,14]. With clavicular shortening greater than 15 mm, changes in the length-tension relationship causes the shoulder girdle’s musculature to lose its mechanical efficiency, resulting in decreased shoulder strength [13]. In an *in-vivo* evaluation, this weakness affected extension, internal rotation, and adduction. Additionally, a shortened clavicle increases anterior scapular tilt, resulting in altered glenohumeral and scapulothoracic kinematics akin to pathologic scapular dyskinesia [15,16].

However, when the scapulothoracic kinematics associated with a shortened clavicle have been examined, these investigations have relied on manual manipulation to achieve forward elevation [17,18]. Nevertheless, they illustrate the limited scapular external rotation and posterior tilting that occur with a shortened clavicle.

In order to better understand how clavicular malunion affects shoulder kinematics in throwing athletes, we conducted a biomechanical study using a validated robotic system to assess abduction (ABD) and an abbreviated throwing motion (ATM) [19]. We compared the shoulder's kinematics with an intact clavicle, a simulated clavicle fracture, a simulated malunion with 2 cm of shortening, and a plated clavicle (after open reduction-internal fixation (ORIF)). *We hypothesized that simulated clavicle malunions with 2 cm of shortening would significantly alter the glenohumeral (GH) joint kinematics*, *and that plate fixation of the clavicle fracture would restore the GH kinematics to baseline*.

## Materials and Methods

### Testing Apparatus

**Fig 1:**
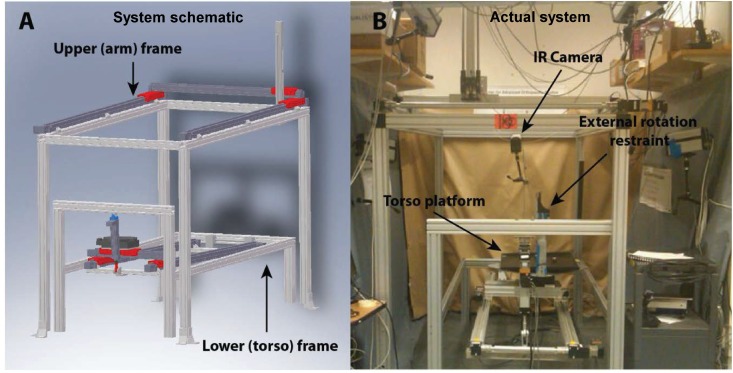
**[A]** Cadaveric system for simulation of real time biomechanics of the shoulder motion—Schematic representation of the testing system high highlighting the upper (torso) and lower (hand frames); **[B]** Actual frame displaying IR camera positioning, torso platform, and cadaver restraint

An automated upper extremity testing system was used to precisely move the arm in 3D space based on prescribed motion trajectories. This system has been validated and used in a number of studies *apriori* [15,19–22]. It encompasses a lower frame (**Fig 1A**), which houses an intact cadaveric torso, and an upper extremity frame, which controls the upper extremity to affect a programmed motion trajectory (**Fig 1B**). The torso frame allows movements in the x-, y-, and z-axes with rotation around the z-axis. The upper extremity frame allows movement in in the x-, y-, and z-axes. All seven degrees-of-freedom are controlled using actuators and a feedback system via a centralized controller. Programmable software can generate a precise motion trajectory reproducibly and accurately within the limits of the actuators, where the coefficient of variation is less than 0.5% for all axes. The absolute and percent errors in the displacement of all axes were 0.1% and 0.5% respectively [19].

### Cadaveric Specimens

Fresh-frozen human cadaveric torsos were acquired from Medcure Anatomical Tissue Bank (Portland, Oregon, USA). Three torsos from Caucasian males with an average age of 55 ± 4 years, height of 190 ± 4 cm, and body mass index (BMI) of 27.1 ± 1.85 kg/m^2^ were used for this study. Both shoulders were tested on each specimen for a total of six shoulders. Torsos were mounted on a rod and foam fixture, as previously described, and a Schanz pin was inserted through the distal radius and ulna after the hand was disarticulated [15]. For each shoulder, the skin and the deltoid muscle were removed to access bony structures. Reflective marker clusters were placed in the humeral shaft, the posterolateral acromion, and the sternum [23].

### Simulation of Throwing Motion, Abduction and Implementation of Clavicular Testing Conditions

**Fig 2:**
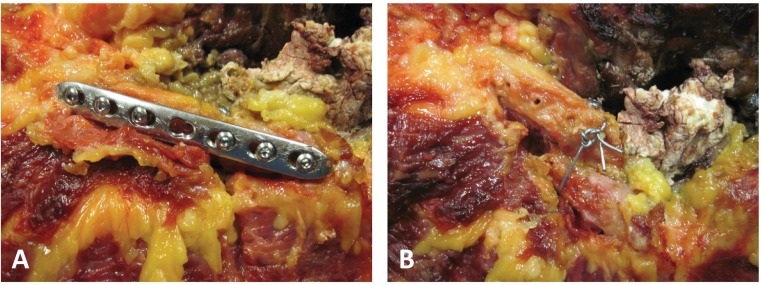
**[A]** ORIF to restore length using 7-hole neutralization plate, where the lag screw can be seen above the plate; **[B]** Exposed clavicle with trans-osseous wire fixation maintaining a rigid 2cm bony overlap

Testing was completed for four different conditions: intact clavicle/baseline (BL), midshaft clavicle fracture (CLF), open reduction-internal fixation of the fractured clavicle (ORIF), and clavicle malunion with 2 cm of shortening (MAL). The clavicle fracture was created using an oscillating saw to sever the clavicle obliquely at its midpoint. ORIF was conducted according to AO standards (Arbeitsgemeinschaft für Osteosynthesefragen, Davos, Switzerland) using a lag screw, placed across the oblique fracture, and an anterior 7-hole, 3.5 mm COMBI plate using 3 bicortical 3.5 mm compression screws on each side of the fracture (the screws closest to the fracture were approximately 0.5 mm from the plane of the fracture; **Fig 2A**). Finally, malunion was created by placing a lag screw through the two fragments with two cm of overlap (Bayonnette opposition). A cerclage wire was added for supplemental fixation (**Fig 2B**). Throughout testing, the specimens were kept moist with physiologic 0.9% saline, and testing room temperature was maintained at 24°C.

**Fig 3:**
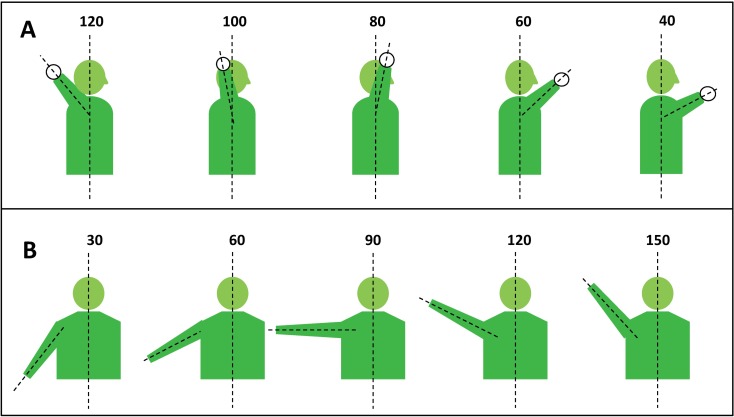
**[A]** Lateral diagram highlighting abbreviated throwing motion (ATM) with humerus maintained at 90° abduction; **[B]** AP diagram for range of abduction (ABD). Data was collected continuously throughout fluid motion

For each condition, three repetitions of the ATM and ABD motions were performed in sequence without resting between repetitions to limit hysteresis. For ATM, the humerus was placed at 90° of abduction with the elbow at 90° of flexion. To simulate the transition from late cocking to acceleration, the upper arm was held against an external restraint while the humerus was externally rotated to 120°. The throwing motion was then created by internally rotating the arm 80° (from 120° of external rotation to 40° of external rotation) (**Fig 3A**). For ABD, the arm was lifted 120° in the plane of the scapular body from 30° of ABD to 150° of ABD (**Fig 3B**). Throughout this arc, the arm was held in neutral rotation.

### Motion Analysis

Five Qualisys ProReflex (Qualisys AB, Gothenburg, Sweden) high-speed cameras (120 Hz) were used to collect the motion of the passive retro-reflective marker clusters embedded into the humeral shaft, the sternum, and the acromion. The positioning of the marker clusters has been reported previously [15,21]. Anatomical landmarks were used to calibrate the reference frame with respect to the technical [bone-embedded] marker clusters using a pointed-wand in accordance with the International Society of Biomechanics (ISB) guidelines [23]. The fully calibrated system can detect movements greater than or equal to 0.3 mm. The motion of each segment and the instant center of rotation of the GH joint were calculated within the scapular reference system [24]. The exact angles of shoulder abduction and external rotation (arm position) were recorded as an independent variable using a digital inclinometer (US Digital, Vancouver, WA, USA) and used to confirm the motion achieved. The x-axis, y-axis, and z-axis corresponded to anterior-posterior (AP), superior-inferior (SI), and medial-lateral (ML) planes, respectively.

### Statistical Analysis

Motion was recorded continuously from ABD 30° to ABD 150° and ATM 120° to ATM 40° throughout the three repetitions for all four conditions. Absolute GH translation was calculated for each condition (BL, CLF, MAL, ORIF) at each axis. Generalized Estimating Equations (GEE) analysis was performed to compare GH translation for all conditions on each axis and motion trajectory. Total translation was calculated for each condition between ABD 30° - 150° and ATM 120° – 40° using the distance formula.

With six specimens from three donors included (three pairs), a statistical power of 80% allowed for detection of a difference of greater than 1.0 mm of GH translation and 85% power to detect mean differences of greater than 1.2 mm of translation using GEE with a compound symmetry correlation structure to handle the paired specimens (nQuery Advisor, Statistical Solutions, Boston, MA, USA). Statistical analysis was conducted with SPSS (version 21.0, IBM-SPSS, Armonk, NY, USA). Two-tailed P values less than 0.05 were considered significant.

## Results

**Fig 4:**
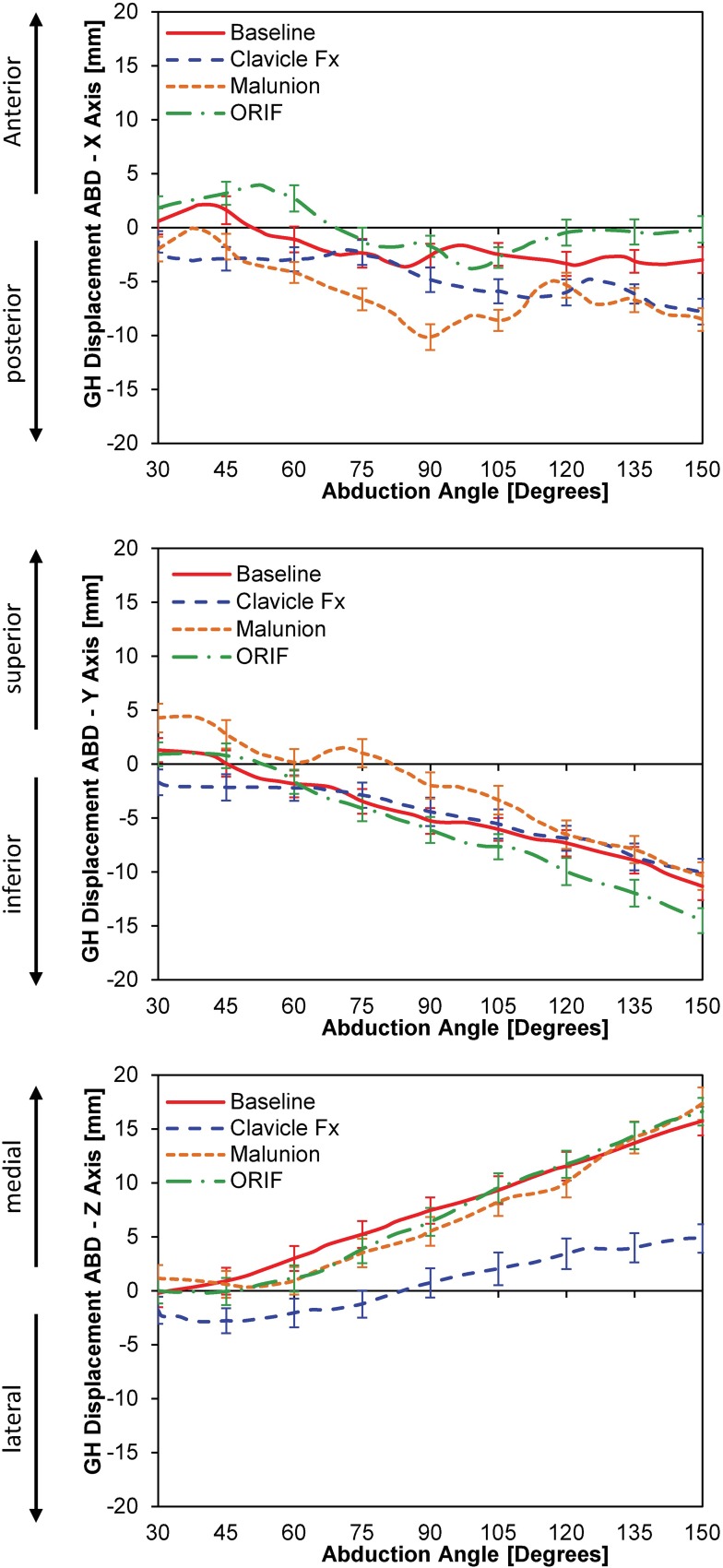
**[A]** Glenohumeral translation during abduction; **[B]** Translation in the x-axis (anterior-posterior); **[C]** Translation in the y-axis (superior-inferior); and Translation in the z-axis (medial-lateral)

Clavicle fracture (CLF) resulted in significant increase in posterior translation (x-axis) compared to BL at 45°, 105°, 120°, 135° and 150° of abduction (**Fig 4A**). Malunion resulted in significant increase in posterior translation compared to baseline at 45°, 60°, 75°, 90°, 105°, 135° and 150° of abduction (**Fig 4A**). Plate fixation (ORIF) resulted in significant anterior translation compared to BL at 60°, 120°, 135° and 150° of abduction (**Fig 4A**). Following ORIF, GH translation was different from CLF and MAL at all degrees of abduction (**Fig 4A**) except for 75° for CLF (p = 0.18).

GH translation in the SI plane (y-axis) demonstrated significant differences between BL and MAL at 30°, 45°, 75°, 90°, and 105° of abduction (**Fig 4B**). No differences were observed between BL and ORIF between 30° and 105° of abduction (all p > 0.05; **Fig 4B**). However, at the end of abduction, ORIF demonstrated a significant increase in inferior translation compared to BL at 120°, 135°, and 150° of abduction (**Fig 4B**).

In the ML plane (z-axis), no statistical differences in GH translation were observed among BL, MAL and ORIF conditions for any degree of abduction (**Fig 4C**). However, clavicle fracture (CLF) resulted in more lateral translation during abduction at 45°, 60°, 75°, 90°, 105°, 120°, 135°, and 150° (**Fig 4C**). ORIF did not significantly alter GH translation when compared to MAL in the ML plane (**Fig 4C**).

**Fig 5:**
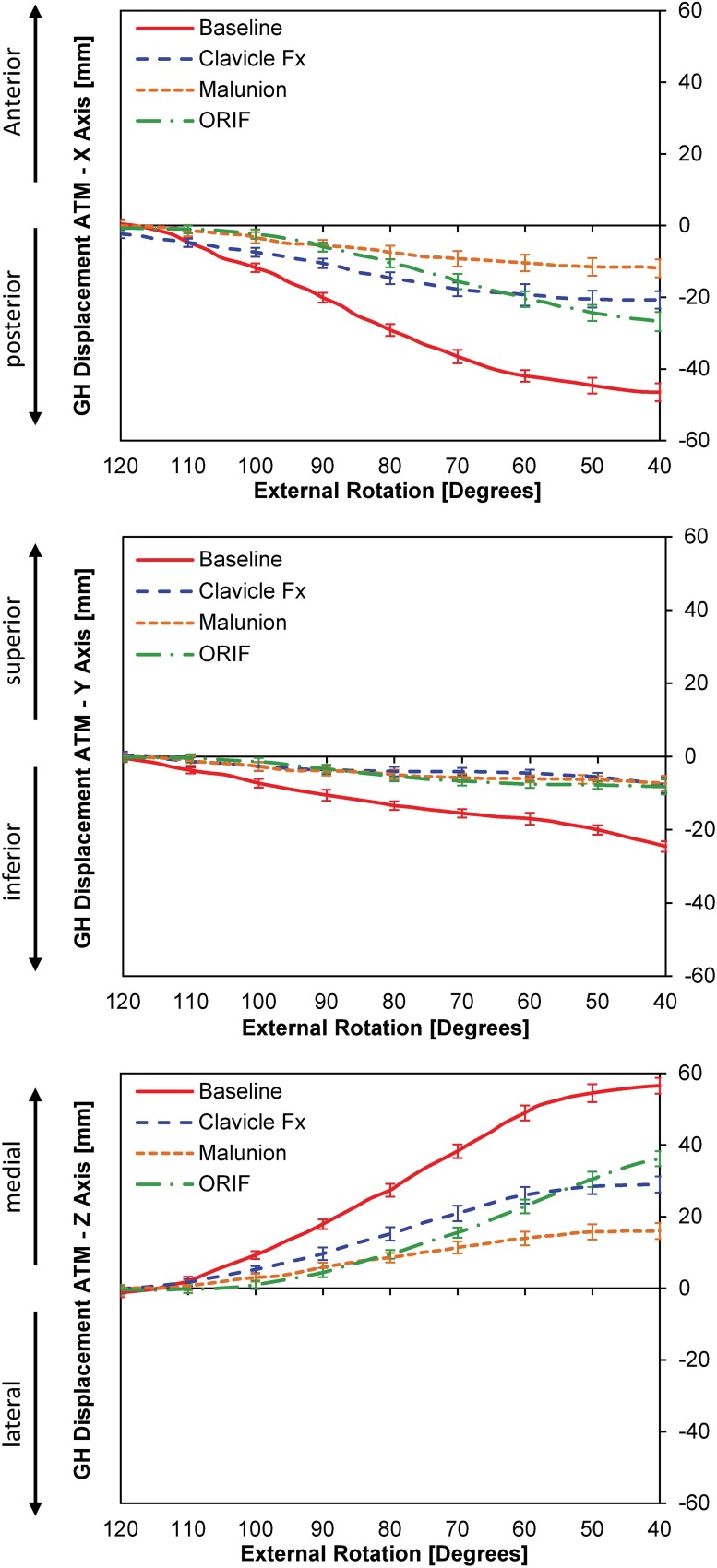
**[A]** Glenohumeral translation during an abbreviated throwing motion (ATM); [**B]** Translation in the z-axis (anterior-posterior); **[C]** Translation in the y-axis (superior-inferior); and Translation in the z-axis (medial-lateral)

For ATM, CLF, MAL, and ORIF all significantly increased anterior translation relative to BL at 100°, 90°, 80°, 70°, 60°, 50°, and 40° of external rotation (**Fig 5A**). Both ORIF and CLF were significantly more posterior than MAL at 70°, 60°, 50° and 40° degrees of external rotation (**Fig 5A**). ORIF did not restore normal kinematics in the AP plane.

ATM also resulted in a significant increase in superior translation following CLF, ORIF, and MAL at 110°, 100°, 90°, 80°, 70°, 60°, 50° and 40° of external rotation (**Fig 5B**). As was the case in the AP plane, ORIF did not restore the GH kinematics to baseline.

For ATM, less medial translation was found relative to BL for CLF, MAL and ORIF from 100° to 40° of external rotation (**Fig 5C**). MAL had significantly more lateral translation than ORIF from 70° to 40° of external rotation (**Fig 5C**).

## Discussion

To the best of our knowledge, this study is the first to assess the effects of clavicle fracture, malunion, and length restoring plate fixation on GH kinematics during passive abduction and abbreviated throwing motion. Our study aimed to examine how clavicle pathology alters GH kinematics due to scapular protraction and increased anterior tilt [17,18,25]. Our technical ability to simulate abduction and an abbreviated throwing motion using a robotic upper extremity testing system allowed us to explore these conditions.

Recent clinical studies have suggested that operative management of midshaft clavicle fractures improves performance in athletes [26]. Despite the potential complications of surgery (hardware irritation, screw migration, pin migration, peri-incisional numbness, and refracture), rigid fixation is believed to improve functional outcomes, decrease the rate of nonunion, and expedite return to activity [26]. Previous studies have documented that a shortened clavicular malunion does not limit range of motion [17,27,28]. However, there is concern that malunion may result in decreased strength [13], limiting an athlete’s competitive edge.

This loss of strength can be explained by how the change in position of the clavicle results in a change in the scapula, altering GH function [13,14,27,29]. For example, clavicular shortening reduces the moment arm of the pectoralis major, thereby weakening this important forward flexor [17,25]. Similarly, increased anterior translation of the humerus during ATM effectively weakens the deltoid during the late cocking phase of throwing by shortening its force-tension relationship [30].

The data from this study demonstrate increased superior and posterior translation of the humerus in relation to the glenoid when comparing MAL to baseline in abduction. Shortened moment arms of the posterior deltoid, teres minor, teres major, subscapularis, infraspinatus, and latissimus dorsi from posterior translation of the humerus may help explain the extension weaknesses reported by Ledger et al [13].

In addition to restoring strength, minimizing aberrant GH contact pressures and translations should be prioritized when treating fractures to prevent unwanted long term sequelae [31–34]. The superior shift of the humerus caused by MAL during both ABD and ATM effectively narrows the subacromial space, increasing the risk of impingement and shear force that may accelerate rotator cuff degeneration [33,34]. Importantly, these changes were seen at angular positions consistent with daily activity. Furthermore, a change in orientation of the humeral head on the glenoid may alter joint contact forces. This shear may place a patient at higher risk for labral tearing and accelerate osteoarthritic changes [31,32].

Interestingly, ORIF of the fractured clavicle failed to restore normal GH kinematics in the AP plane and displaced the center of rotation of the GH joint in abduction angles greater than 120° and during abbreviated throwing motion from 100° to 40°. The anterior translation of the humeral head may increase the strain on the anterior capsule, increasing an athlete’s risk of injury. While restoring strength is imperative for athletes, clinicians should be wary of alterations in the glenohumeral kinematics after ORIF.

It is noteworthy that none of the cadaveric specimens used for this biomechanical study benefited from clavicular attachments of the sternocleidomastoid muscles. The pectoralis major insertions on the clavicle were detached an additional 2 cm on each side of the fracture to allow for the bony overlap of the MAL condition. These muscles may be important to GH kinematics in abduction and ATM studies. Our study did not simulate the dynamic muscle forces involved in ABD and ATM [35], and as with previous investigations [15,21], glenohumeral joint translation was calculated based on a regression analysis of the instant center of rotation. This estimation relies on an anatomical area landmark, which depend on calibrations that vary among specimens [36]. Also, hysteresis is a source of variability, and in order to minimize the change in tissue elasticity, a uniform testing environment was employed, maintaining tissue moisture, controlled room temperature and data acquisition without delay.

Shortened clavicle malunions are associated with significant increases in posterior and superior glenohumeral translation throughout abduction. Plate fixation failed to restore normal GH motion. This study illustrates the complex interplay of the clavicle and the GH joint. While abnormal clavicle alignment alters shoulder motion, restoration of clavicle length does not necessarily restore GH kinematics to BL. Rehabilitation of the injured shoulder must address the osseous injury and the dynamic forces of the shoulder girdle.
